# Molecule(s) of Interest: I. Ionic Liquids–Gateway to Newer Nanotechnology Applications: Advanced Nanobiotechnical Uses’, Current Status, Emerging Trends, Challenges, and Prospects

**DOI:** 10.3390/ijms232214346

**Published:** 2022-11-18

**Authors:** Riaz A. Khan, Hamdoon A. Mohammed, Ghassan M. Sulaiman, Amal Al Subaiyel, Arjunan Karuppaiah, Habibur Rahman, Sifiso Makhathini, Poornima Ramburrun, Yahya E. Choonara

**Affiliations:** 1 Department of Medicinal Chemistry and Pharmacognosy, College of Pharmacy, Qassim University, Qassim 51452, Saudi Arabia; 2 Department of Pharmacognosy and Medicinal Plants, Faculty of Pharmacy, Al-Azhar University, Cairo 11751, Egypt; 3 Division of Biotechnology, Department of Applied Sciences, University of Technology, Baghdad 10066, Iraq; 4 Department of Pharmaceutics, College of Pharmacy, Qassim University, Qassim 51452, Saudi Arabia; 5 Department of Pharmaceutics, Karapagam College of Pharmacy, Coimbatore 641032, TN, India; 6 Department of Pharmaceutics, PSG College of Pharmacy, Peelamedu, Coimbatore 641004, TN, India; 7 Wits Advanced Drug Delivery Platform (WADDP) Research Unit, Department of Pharmacy and Pharmacology, School of Therapeutic Sciences, Faculty of Health Sciences, University of the Witwatersrand, 7 York Road, Parktown, Johannesburg 2193, South Africa

**Keywords:** ionic liquids, nanobiotechnology, nanotechnology, nano-preparation, separations, gene delivery, drug delivery, bioreactors, nano-capacitors, nano-biocatalysis, industrial applications

## Abstract

Ionic liquids are a potent class of organic compounds exhibiting unique physico-chemical properties and structural compositions that are different from the classical dipolar organic liquids. These molecules have found diverse applications in different chemical, biochemical, biophysical fields, and a number of industrial usages. The ionic liquids-based products and procedural applications are being developed for a number of newer industrial purposes, and academic uses in nanotechnology related procedures, processes, and products, especially in nanobiotechnology and nanomedicine. The current article overviews their uses in different fields, including applications, functions, and as parts of products and processes at primary and advanced levels. The application and product examples, and prospects in various fields of nanotechnology, domains of nanosystem syntheses, nano-scale product development, the process of membrane filtering, biofilm formation, and bio-separations are prominently discussed. The applications in carbon nanotubes; quantum dots; and drug, gene, and other payload delivery vehicle developments in the nanobiotechnology field are also covered. The broader scopes of applications of ionic liquids, future developmental possibilities in chemistry and different bio-aspects, promises in the newer genres of nanobiotechnology products, certain bioprocesses controls, and toxicity, together with emerging trends, challenges, and prospects are also elaborated.

## 1. Ionic Liquids (ILs)–An Introduction: Structures, Functions, Outreach, and Scope

**Figure 1:**
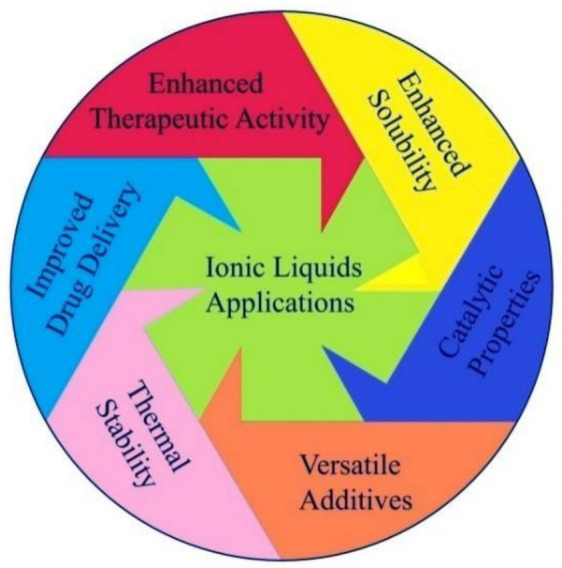
Representation of the general applications of ILs.

Recent years have seen a great deal of interest and anticipation in nanoscale materials, the building blocks of nanoscience and nanotechnology-based products and procedures, due to the large surface area and unique chemical and physical properties of the nanoscale entities [1]. On other hand, there is also a growing interest in the potential uses of ionic liquids (ILs) in biomedicine, biocatalysis, bioseparations, green bio-composites, biosensors, fluid thermodynamics, catalysis, organic synthesis, electrochemical windows, separation, purification, gas sensors, ion sensors, biomass processing, and functional energy production [2,3,4]. ILs applications have been achieved through the functional properties of ILs (Figure 1), and this has also provided crucial advances in creating novel pharmaceutical products, and has opened newer avenues in newer medical strategies towards procedures, processes, and product development [5].

In simpler terms, ILs are defined as ionic compounds which are liquids below 100 °C [6]. They are typically composed of large asymmetric organic cations and inorganic, or organic anions with distinct properties, such as high viscosity and very low vapor pressure under ambient conditions, making them non-volatile. Among other properties, i.e., thermal stability and miscibility, are primarily determined by the anionic part of ILs molecules, whereas viscosity, surface tension, and density factors are determined by the length of the alkyl chain in the cation part of the molecule, and/or also the shape and symmetry that contributes to these properties of viscosity, surface tension, and density, owing to the structural contributions of the ensuing shape and symmetry of ILs [7,8]. Due to these unique properties, ILs have been used extensively in a wide variety of applications at molecular assembly levels for functions and applications in product formation, as well as in procedures development. Table 1 lists several applications of ILs, their associated functions, and the commonly used ILs for the purpose.

**Figure 2:**
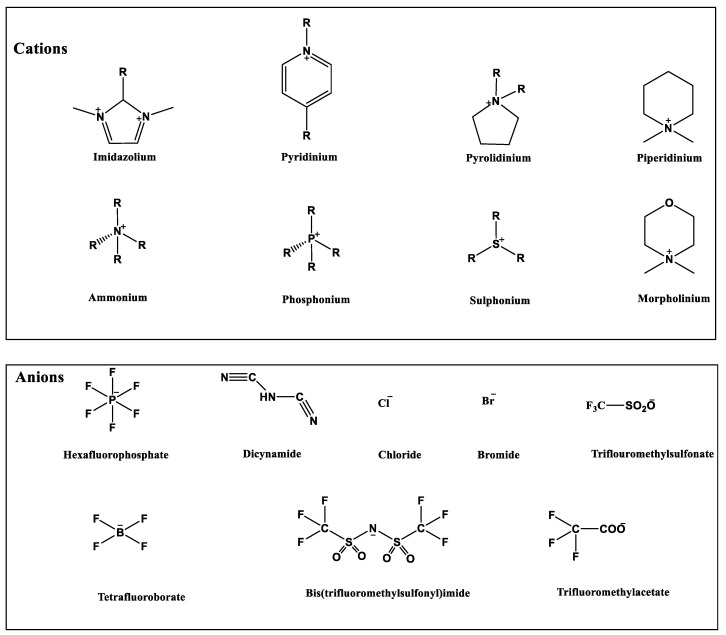
Names and chemical structures of the most studied IL cations and anions.

The most common ILs anionic constituents are polyatomic inorganic species, i.e., PF 6 and BF 4, whereas the most common cations are pyridinium and imidazolium ring species with one or more alkyl groups attached to the nitrogen or carbon atoms (Figure 2) [23]. Considering both the properties of ILs, and nanosystems (NS) presented individually, their combination is a means of achieving more efficient hybrid systems that share the properties of both materials, particularly for their potential applications in various fields, including the pharmaceutical sector that also includes product development for drugs, genes, and other payload delivery systems [6]. The therapeutic efficacy of the drug is defined, among other parameters, by its solubility and bioavailability [16]. Several pharmaceutical drugs are sparingly soluble in aqueous media, and it does not help to achieve their pharmacological effects. To overcome the solubility limitations, ILs have been investigated as an alternative to classical organic solvents as solubilizing media. The solubilizing ability of ILs has been demonstrated to increase the aqueous solubility and their water dispersion of many pharmaceutical products for various purposes other than therapeutic. The solubility of drugs have also been found to increase [16]. A previously reported study evaluated the solubility of ibuprofen and compared it with conventional hydrotropes. The obtained results of the reported study had clearly shown the enhancement of ibuprofen solubility within ILs. ILs [C_4_C_1_im][SCN] and [C_4_C_1_im][N(CN)_2_] were found to be most promising for the solubility enhancement purposes of ibuprofen. The authors observed 60- and 120-folds increased solubility with ILs concentrations of *circa* 1 mol kg^−1^, respectively [24]. Therefore, the synergistic effects of integrating ionic liquids and nanotechnology for the purpose of developing attractive and efficient nanomaterials for industrial applications have been at the forefront of IL-based materials and procedure advancements.

### 1.1. ILs Emerging Applications in the Nanosciences Fields

**Figure 3:**
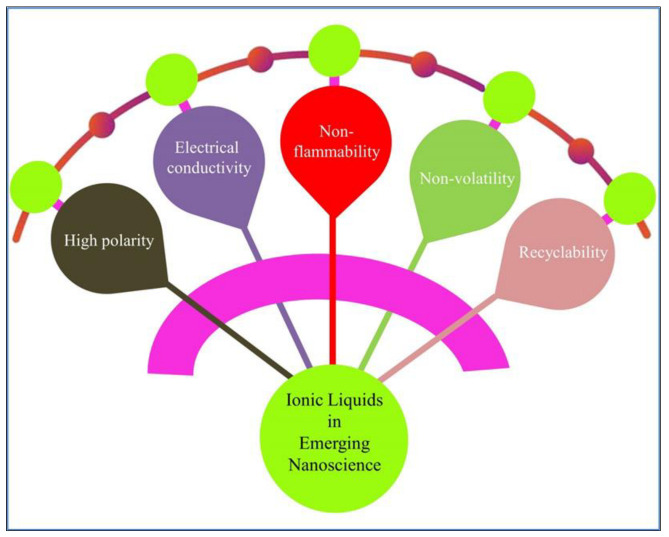
Ionic liquids and their pertinent properties for the emerging applications in nanosciences fields.

In recent years, nanoscale materials have attracted a great deal of interest due to their extremely high surface area and chemical and physical properties that distinguishes them from bulk phase and individual molecules [25]. Combining ILs with nanotechnologies offers more appealing and effective possibilities for future industrially useful nanomaterials of various natures including the pharmaceutical and biotechnical products equipped to deal at nanoscale dimensions. In addition to the excellent physicochemical properties (Figure 3), ILs can also act as a strengthening and stabilizing agent for nanomaterials, thereby enabling the creation of new multi-dimensional nanomaterials. Currently, researchers have shown success in using ILs for improving the thermal and electrochemical stability of novel nanoparticles (NPs), nanocomposites, and nanomaterials. ILs could be used in nanocomposite materials for material interaction and to assist in electron transfer reactions, giving rise to new electro-chemical and materials’ strengthening properties. Materials such as these are projected to find applications in certain pharmaceutical-based products, also employing the electro-sensors, electronics, and photonics [26]. In addition to improved thermal and electrochemical stability, particle size control, increased conductivity, reduced toxicity, and increased reactivity, ILs in nanomaterials are benign in nature and environmentally friendly. In essence, ILs allow nanotechnology to take on a broader range of applications due to their ability to improve existing materials. The field of nanotechnology is one of the most exciting and important areas in modern science, wherein nanomaterials can be applied to a wide range of research areas, including electro-analytical procedures and product development for nanoscale machination, drug delivery, auto-synthesis, controlled catalysis, products and bio-analytes analysis, and chemo-bio sensors capable of detecting and quantifying at the nano- and sub-nano-scale owing to their high sensitivity, specificity, and selectiveness [27]. Because of the intriguing properties of ILs, researchers have created novel ILs modified functional nanomaterials, which have given rise to opportunities to further develop new nano-scale and bulk-scale materials. These newly designed functionalized nanomaterials have been created by using ILs as synthetic mediums, precursors, templates, and components. Furthermore, the influence of ILs in the synthesis of NPs involves maintaining the size of nano-particles, which reduces aggregation and particle size distribution while significantly improving the materials’ conductivity, solubility, and catalytic activity [1].

### 1.2. ILs: Structure, Composition, and Physicochemical Properties

**Figure 4:**
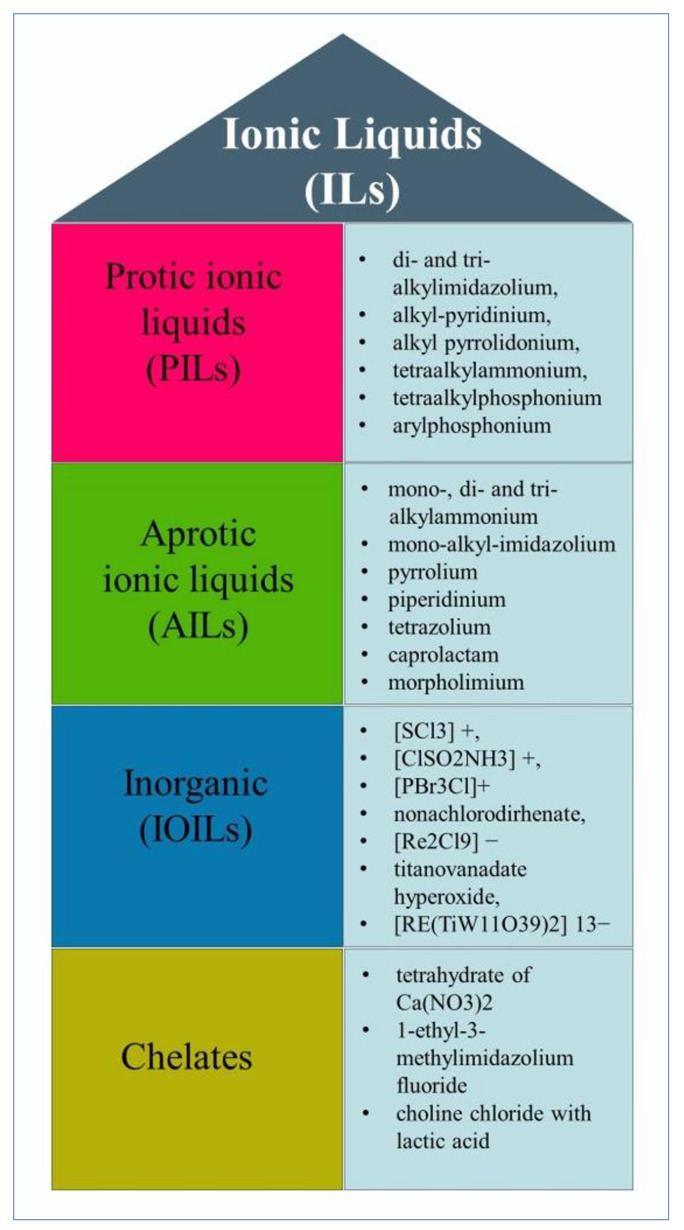
IL types and their classifications.

In terms of the structural property-based functional characteristics, ILs have created huge demands in modern-day chemistry, biochemistry, and nanotechnology-based industries as well. Typically, ILs are highly stable materials, and provide a high boiling point. Additionally, it possesses good electrochemical and catalytic properties. It can also be used to stabilize proteins, and facilitate enzymatic reactions [28]. ILs are classified as protic ionic liquids (PILs), aprotic ionic liquids (AILs), inorganic (IOILs), and chelates-based ILs (Figure 4). Each IL differs in terms of its physico-chemical properties, functional variability and activity levels, function types, and strength of the directional and non-directional forces between the ions of ILs, that give them the unique properties ILs hold [21,29].

The chelate complexation between coordinating solvent and salt produces properties similar to typical ILs. The Kamlet–Taft parameters, Gutmann acceptor numbers and chelating effects studies have been carried out for several atomic nuclei by NMR spectroscopy. The solvating and low/non-volatility properties have been utilized for conducting many organic transformations and reactions, i.e., Aldol, Kabachnik–Fields, and Diels–Alder. ILs role in expanding Boron Chemistry applications is also emerging [30]. These chelates-based ILs are also termed as SILs (Solvate Ionic Liquids) [31]

The low melting point, better solubilization strength, high thermal stability, high flash point, high polarity, low viscosity, better flowability, high miscibility with water, high ionic conductivity, electrochemical stability, low vapor pressure, low reactive nature, non-flammability, anti-electrostatic effects, air and water stability, and their characteristics, such as ‘designer solvent’, through structural manipulations/replacements of cation and/or anionic parts of the structure to tailor the intended properties, have placed ILs in the designer range of solvating materials which can be transformed for the intended properties according to the specific need for a solvent wherein different properties are required to carry out different processes, procedures, reactions, nano-materials preparations, and product development. The designer capacity has afforded the chiral ILs, suitability for stereo-controls and chirality induction in enantioselective transformations towards processes and products development. 1-Ethyl-3-methylimidazolium L-(+)-lactate and 2-hydroxyethyltrimethylammonium L-(+)-lactate are two ionic liquids with chiral anions, while 2-hydroxy ethyl trimethyl ammonium-L-(+)-lactate and achiral 2-hydroxy ethyl trimethyl ammonium acetate have been termed as natural ILs owing to the presence of natural ions in their structures [32]. The short-chained quaternary ammonium and phosphonium dialkyl phosphates, i.e., tributyl methyl ammonium, tributyl methyl phosphonium, triethyl methyl ammonium, and triethyl methyl phosphonium dibutyl phosphates have shown typical IL properties together with no hydrolysis, and stability towards strong bases since no acidic protons are present in their structures, unlike the imidazolium-based ILs. These ILs also have different solvating properties since there are no aromatic p-electrons, or any free lone-pairs of electrons on the cationic structure which results in fewer donor–acceptor interactions with solutes. Nonetheless, ILs remain a more desirable solvent than conventional organic solvents of volatile and non-volatile nature. The functionalized ILs are emerging as next-generation solvents and nano-utility material [33,34]. The fluorinated ionic liquids (FILs) containing the fluorinated chain lengths equal or greater than four carbon atoms have shown modified properties as compared to the conventional ILs [35]. ILs as components in fluorescent functional materials [36], temperature-responsive micro-emulsion derived from tropine ILs [37], cationic surfactants-like ILs [38], together with the regulatable interfacial property and flow behavior of ILs confined in graphene nanochannels have been studied [39].

The critical properties, critical temperature, critical pressure, and acentric factors of a number of ILs, which are not possible to detect owing to the decompositions of ILs before they reach their critical state, were determined through vapor-liquid equilibrium data of binary mixtures of CO2+IL, and the Peng–Robinson equation of state (PR EoS), together with Soave–Redlich–Kwong equation of state, bubble-point pressure, and differential evolution optimization methods were used [40]. ILs used were 1-ethyl-3-methylimidazolium hexafluorophosphate ([emim][PF6]), 1-ethyl-3-methylimidazolium bis(trifluoromethylsulfonyl) imide ([emim][Tf2N]), 1-butyl-3-methylimidazolium tetrafluoroborate ([bmim][BF4]), 1-butyl-3-methylimidazolium hexafluorophosphate ([bmim][PF6]), 1-butyl-3-methylimidazolium bis(trifluoromethylsulfonyl) imide ([bmim][Tf2N]), 1-hexyl-3-methylimidazolium tetrafluoroborate ([hmim][BF4]), 1-hexyl-3-methylimidazolium hexafluorophosphate ([hmim][PF6]), 1-hexyl-3-methylimidazolium bis(trifluoromethylsulfonyl) imide ([hmim][Tf2N]), 1-octyl-3-methylimidazolium tetrafluoroborate ([omim][BF4]), and 1-octyl-3-methylimidazolium hexafluorophosphate ([omim][PF6] [38].

## 2. Primary and Advanced Applications of ILs: Current Status, Challenges, and Prospects

The major challenges in the primary and advanced applications of ILs have been their viscosity, thermal capacity, and the purity. Techniques development for attaining purity is crucial. ILs structural variability through maneuverings of the anionic and cationic parts of their structures have produced a number of IL variants with differing physico-chemical properties, and have contributed to ILs of different viscosity and thermal capacity. The control of physical and chemical properties had been achieved through structural manipulations, and quality by design approaches which are emerging through various permuted combinations of the anionic and cationic structures interventions. The structural modification exercises have provided newer classes of ILs, including chiral ILs suitable for chiral applications in product and procedure developments. The challenges faced in the early ILs’ properties-based applications have, to large extent, been overcome, and specific ILs with definite physico-chemical properties are constantly under development. Towards further development in ILs applications, their solubility have immensely contributed, and still nano-scale and quantum-levels materials, and drugs needs fresh look for solubilization and through it attaining better stability and shelf-life from ILs that are liquid at room temperature. Moreover, combining two drugs for combination therapy through active common ions formulation in ILs seems the way forward for producing effective dose regimens. Among other challenges, ILs use as chromatographic solvent and extractant needs further development, and is expected to rise to the extraction capabilities equivalent to the super critical fluid made up of several combinations, including CO_2_ and water, and the ongoing efforts towards increasing CO_2_ solubility holds further promise.

### 2.1. ILs as Nanotechnical Solvents in Pharmaceutical Products, Procedures, and Processes

Typically, based on the charge density of the anions, ILs will act either as a hydrophilic, or a hydrophobic entity in water. Hence, it can react with both polar and non-polar solutes [29]. ILs have been reported as having high potential and versatility in terms of chemical structure design towards target applications in the field of pharmaceutics [16,28]. ILs have high polarity, electrical conductivity, extremely low values of their saturated vapor pressure, non-flammability, non-volatility, and recyclability [41]. ILs are a suitable replacement for volatile organic solvents (VOCs) in various fields, such as preparative steps of nano and non-nano materials formation/formulation, chemical synthesis and their catalytic controls, electrochemical processes and products for various uses, biotechnology and pharmaceutical products, etc. [28,29]. ILs are widely used in analytical chemistry for the extraction and separation of, primarily, bioactive compounds [21]. Towards the development of pharmaceutical formulations, physicochemical properties, such as solubility, bioavailability, permeation, polymorphism, and stability demand an effective solution for the achievement of therapeutic effects of the drug. The combination of ILs and nanoparticles (NPs) is highly appealing as a nanotech solvent (Figure 3), but it has been reported to have limited colloidal stabilization which provided initial direction towards their employment [42]. ILs have been investigated as solvents, reagents, and anti-solvents in the synthesis and crystallization of active pharmaceutical ingredients (APIs). It has been used as solvents, co-solvents, and emulsifiers in preparing liquid formulations, and development and improvement of drug delivery systems [16]. ILs have the ability to dissolve a number of chemical substances and, therefore, are preferred as solubilizing agents in the preparations of various NPs-based formulations [42]. Previous studies suggested that thioamide-derived ionic liquids serve as a modular platform for lipid-mediated gene delivery [43]. Moreover, ILs with surfactant behavior, referred to as surface-active ionic liquids (SAILs), displayed high potential to increase the dissolution of a number of pharmaceutical agents, based on micellar support in aqueous media [44].

### 2.2. Nanocrystals Formations

Technically, a good solvent system inhibits the nanoparticles’ aggregation and disperses the particles to form a stable colloidal suspension. The long-chain hydrocarbons with a surface-binding head group have been employed as ligands to disperse nanocrystals (NCs) in non-polar organic solvents. Solvents have also played crucial roles in nucleation and growth dynamics during NCs synthesis. However, synthesizing hard-to-crystallize inorganic phases in a nano-crystalline form is limited by the inability of the currently available solvents to reach the desired crystallization temperatures [45]. Several studies have reported on the application of ILs in APIs crystallization as adjuvants. ILs are not only used for the development of new polymorphic forms, but they have also been used to manipulate the crystal form to have enhanced properties, and ultimately to separate and isolate the specific polymorphic forms that are not achievable through conventional solvents [16]. IL-based crystallization techniques, which included solvent–anti-solvent, cooling crystallization, and drowning-out techniques, have been proposed to enhance the physico-chemical properties and polymorphic forms of various pharmaceutical ingredients. In a previously reported study, cellulose nanocrystals were developed from microcrystalline cellulose. ILs solvents, 1-ethyl-3-methylimidazolium chloride [EMIM][Cl], and 1-propyl-3-methylimidazolium chloride [PMIM][Cl], were utilized. The dynamic light scattering (DLS) results indicated that the smaller particle sizes were obtained when the start-up material was treated with [PMIM][Cl] as compared to the [EMIM][Cl] use when material was hydrolyzed [41]. Nanocrystals synthesized using the ILs, 1-butyl methyl pyrrolidinium bis (trifluoromethanesulfonyl) amide ([Bmpyr][Tf_2_N]) and/or 1-butyl-1-methylpyrrolidinium trifluoromethanesulfonate ([Bmpyr][TfO]), showed that neither the crystalline structure nor the NPs’ sizes had changed after nanocrystal dispersion in these ILs [46].

### 2.3. Solubilizations, and Self-Assembly of Polymeric Materials in ILs

Solvation is the prime property of ILs, as they were primarily designed to be used as solvents, and the presence of structural domains in ILs greatly influences their solvation capabilities [47]. ILs have been shown as excellent solvents for a wide range of important chemical processes, products, and starting materials, thereby providing new avenues for chemical reactions, and products’ purification, as well as tackling tough and stubborn separations in industry [48]. The study reported by Sedov et al. in 2017 and 2019 demonstrated that the excess chemical potential and limiting activity coefficients for hydrocarbons dissolved in alkylammonium protic ionic liquids (PILs), (propyl- and butyl-ammonium nitrate, butylammonium thiocyanate) are lesser than those observed in the aprotic ILs (AILs) with similar molar volumes of the experimental materials. However, the selectivity in solubilizing the materials, viscosity, and ionic strength as electrolytes are important factors [49,50]. Moreover, micellar transfers between the aqueous phase and hydrophobic natured ionic liquids have also been demonstrated as part of the thermo-reversible action with implications for thermal triggering and controlling of reactions and processes [51]. Furthermore, reversible transfer of a dye-loaded nanocarrier, poly(2-ethyl-2-oxazoline)-*block*-poly(2-nonyl-2-oxazoline) substrate between ionic liquid and water was also demonstrated for phase-change feasibility of payload delivery, stability, and for protection against degradation, payload leak, and deformity, as well as insulation to the loaded material and the carrier against solvating media-based repercussions [52]. Towards more conceptual applications of the thermo-reversibility-based material transfer, preparation of stabilized ionogels through hydrogen-bondings have been recently undertaken involving the amphiphilic oligoether-based ILs and water to produce gels with tunable properties, i.e., ionic conductivity, higher melting temperature and mechanical moduli. The ionogel products were characterized through PMR (proton nuclear magnetic resonance) spectroscopy, rheological properties, ionic conductivity, surface tension, water uptake, DSC (differential scanning calorimetry), and small angle X-ray scattering investigations [53,54].

### 2.4. Quantum Dots Preparations

Quantum dots (QD) are luminescent semi-conducting materials with dimensions smaller than 10 nm, and their physicochemical properties can be easily tuned by controlling both the size of the dots, and how they are synthesized [55,56,57]. Over the years, QDs have found several widespread applications in solar cells, laser devices, light-emitting devices (LEDs), probe apparatuses, and biosensors [58,59]. In addition, as new fluorescent tags in a wide variety of biological and biomedical fields, QDs have emerged as a promising tool in biomedical experimentations, clinical diagnostics, drug delivery, and photodynamic therapy (PDT) [55]. The preparation of QDs has been successfully achieved by many traditional methods which involve volatile organic solvents at high temperatures [60]. However, in order to make QDs more accessible for different applications, new synthetic methods that are both effective and economically viable, are required for preparing QDs. In 2017, Shikha and colleagues reported an economically efficient, and green approach for preparing zinc sulfide (ZnS) QDs, where zinc (metal M) containing ionic liquid (IL), [C_n_mim][ZnCl_3_] (*n* = 4, 8, and 16) was used as a precursor, template, and solvent to prepare the metal ionic liquid, MIL [61]. Using a simple grinding technique, the ZnS QDs were prepared at RT (room temperature). The ZnS QDs synthesized using this method were found to be spherical with an average diameter ranging from 1.5 nm to 5 nm, depending on the length of the MIL alkyl chain. These results suggested that the preparation of QDs and their MIL-dependent characteristics provide a new approach that is simple and economical to manipulate the properties of the nanoscale structures. In another study, a simplified method of preparing photoluminescent (PL) Cu-In-Zn-S QDs (CIZSQDs) was reported [62]. This method involved the use of microwave treatment together with a RT ionic liquid (RTIL). The RTIL, 1-butyl-3-methylimidazoliumtetrafluoroborate [Bmim]BF_4_ was used, which served as a microwave absorbent for both the rapid synthesis and efficient generation of photoluminescent properties (PLP) of the QDs at low temperature. The results from the study showed that the properties of the synthesized CIZS depended on the metal element ratios of the QDs, anion species, and the alkyl chain length of the RTIL. Moreover, the author indicated, that to achieve CIZSQDs with homogenous size distribution, the aggregation could be avoided with the intermediate alkyl chain length of the RTIL. Additionally, coating with a ZnS shell, CIZSQDs provided a high quantum yield (QY) of 24.1%. This technology could be useful for quickly synthesizing numerous QDs with a larger range of emission wavelengths for the PLP and higher QY. These products will have the potential to serve in a variety of applications.

### 2.5. Nano-Structured Coatings, and Films Depositions

Nanomaterials with significantly higher surface-area to volume ratio, have showed significantly higher extraction capacity as compared to other materials used for the similar extraction procedures. The solid phase micro-extraction (SPME) is an excellent example [63]. The use of nanomaterials in SPME coatings has increased dramatically in recent years, and the applications of the nano-structured materials in SPME were reported by Mehdinia and co-workers after successfully preparing polyaniline-based SPME coatings for extraction of polychlorinated biphenyls (PCBs) [63,64]. Nanomaterials are frequently prepared using micro-emulsions, but it has also been demonstrated, that in the presence of a surfactant, ILs can replace water, or any other conventional organic solvent to form novel micro-emulsion systems. Based on the known properties of ILs, the molecules exhibited higher extraction efficiency for a variety of organic compounds and metal ions. As a result, ILs are regarded as potential absorbents for SPME. For example, He et al., successfully prepared an ionic liquid-based SPME coating, and discovered that it had excellent extraction capability for methamphetamine (MAP) and amphetamine (AP) from human urine samples. Meng and Anderson created a polymeric ionic liquid-based SPME coating that demonstrated high selectivity for polycyclic aromatic hydrocarbons (PAHs) extraction capability from aqueous samples. As an SPME coating in a micro-emulsion containing 1-butyl-3-methylimidazolium hexafluorophosphate, a novel nano-structured polyaniline-ionic liquid composite (BPAN) film was electrochemically prepared (BMIPF6) [65]. The novel BPAN coating was then used to extract five organochlorine pesticides through headspace solid-phase micro-extraction (HS-SPME) procedure. The novel BPANI fiber’s properties, such as extraction efficiency, thermal stability, and half-life, were investigated. The effects of various parameters on the amount of OCPs (organochlorine pesticides) extracted from water were investigated. Following that, the optimized method was used to determine OCPs in real water samples.

### 2.6. Formations of Nanoporous Materials

**Figure 5:**
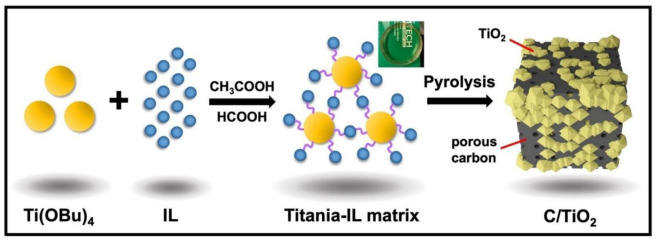
An illustration of C/TiO_2_ composite synthesis via ionothermal synthesis, the purple curved line represents hydrogen bonding between Titania and ILs through non-hydrolytic sol-gel process Reprinted with permission from Ref. [68]. 2022, John Wiley and Sons.

**Figure 6:**
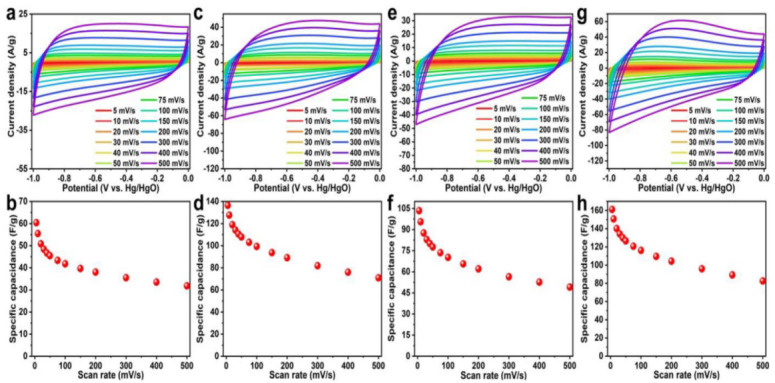
CV profiles and corresponding specific gravimetric capacitances at different scan rates of (**a**,**b**). C/SiO_2_, (**c**,**d**). [CNPIm][NTf_2_]-based C/TiO_2_, (**e**,**f**). C/Nb_2_O_5_ and (**g**,**h**). [CNPIm][NTf_2_]-based pure C, respectively. Reprinted with permission from Ref. [68] 2022, John Wiley and Sons.

Nanoporous materials are classified by the IUPAC based on their pore sizes [66]. The nanoporous materials have a number of beneficial properties that make them suitable for a variety of technological applications, including energy storage, conversion in fuel cells, solar cells, Li-ion batteries, hydrogen storage, and as supercapacitors, in catalysis, sorption applications, gas purification, separation technologies, cell biology, environmental remediation, water desalination, purification, separation, sensors, optical, and electronic and magnetic devices, and drug and gene deliveries [67]. One of the simpler, greener, and scalable methods to fabricate nanoporous materials based on poly(ionic liquid) (PILs) has been reported by Azcune et al., 2014 [66]. The method is based on the radical polymerization of crosslinker-type IL monomers in the presence of an analogous IL that acts as a porogenic solvent. In addition, no waste was generated, and this method was found to overcome certain challenges associated with other concurrent preparative strategies, including the scale up drawbacks, energy consumption (e.g., sintering of hard templates), as well as safety (e.g., the use of harmful volatile solvents). In another study, a facile in situ ionothermal synthetic strategy for preparing highly nanoporous carbon/TiO_2_ nanocomposite supercapacitors was developed (Figure 5) [68]. The method involved the use of ILs as solvent, templating agent, and carbon source to attain nano features, porosity, and a high carbon yield of the final product. The prepared carbon/titanium oxide composite was proposed to have high electrochemical performance as the supercapacitor electrode. The results indicated that the strong interaction of ionic liquids and oxides significantly enhanced the carbon yield of ionic liquids from nearly 0 to 18 wt.%. The C/TiO^2^-4 composite outperformed C/SiO_2_ and C/Nb_2_O_5_ in terms of electrochemical performance as a supercapacitor electrode material due to the synergistic effects of nitrogen-rich carbon species’ high electrical conductivity, and Ti^3+^ self-doping enhanced electrical conductivity of TiO_2_, which delivered a high specific capacitance of 152.4 F g^−1^ at 5 mVs^−1^ with good capacitance retentions of 84.7%, as shown in Figure 6.

### 2.7. ILs in Separation Processes and Fluid Properties

ILs have also been used in a variety of separation applications, including ionic liquid supported membranes, as mobile phase additives, and surface-bonded stationary phases in chromatographic separations, as well as the extraction solvent in samples preparation. This is due to their composition of various cations, and anions in their structures that change the properties, and phase behavior of these liquids, ILs [23]. While ILs have only been recently used in scaled-up and industrial separations, academic interest in ILs is old and growing. Based on tunable IL properties (Table 2), a stable stationary phase for gas chromatography based on alkylimidazolium-based ILs have been developed [69]. It was suggested that these ILs may function as versatile multi-modal media for chromatographic separations due to their unusual selectivity with a “dual nature,” for separating polar compounds, as well as non-polar compounds from the sample’s compositions. In addition, when mixed with other low viscosity solvents, ILs can also be used as mobile phase additives in reverse-phase (RP) chromatographic separations [70,71]. Additionally, ILs have been used to modify the running buffers, as well as to coat capillary walls in the capillary electrophoresis (CE) [71] instrument while operating for better separations. A number of materials, e.g., DNA and their fragments, proteins, peptides, dyes, and polyphenols components have been analyzed by the CE technique without any operational difficulty. Towards maneuvering IL-based separations, the replacement of anion (PF_6_^−^) with (BF_4_^−^) in ILs based on 1-alkyl-3-alkylimadazolium cations, the water solubility of the resultant ILs significantly increased, whereas the anion (Tf_2_N^−^) incorporation was demonstrated to exhibit lesser water solubility. It was further observed that a change in the chain length of 1-alkyl-3-methylimadazolium hexafluorophosphate [CnMIm][PF_6_] caused normally soluble ILs to become very immiscible in water [72], and these properties were of significance in designing the separations utilizing ILs. A number of physical properties, including density, viscosity, melting point, chain length, polar head groups’ presence, structures of the cationic and anionic parts of ILs, and the molecular weights also play part in the separations, and other ILs-based applications in chromatography, extractions, and separations. Table 2 summarizes ILs major use in separation technology.

### 2.8. ILs and Nanoparticles

ILs are a broad group of organic salts with varying structures, and properties which are used in preparation of particles in the nano- and micro-scale ranges [78,79]. Experimentally, ILs are a good solvent for the polymer used in the preparation of NPs, as the polymer swells into the solvent and causes steric repulsion. Another way of stabilization through ILs is based on ion layering of the ILs around the NPs. Adding NPs to ILs can further improve the properties of the resultant system. The addition of charged NPs, for example, can improve the thermo-electric properties of the final preparation, as compared to the use of traditional solvents. A previously reported study explored the key parameters affecting the colloidal stability in dispersions of metal oxide based NPs in ILs [42]. ILs based on imidazolium cations are among the most popular solvents for nano-cellulose preparation, and cellulose decomposition [41]. The most commonly used ILs for dissolving cellulose are 1-butyl-3-methylimidazole chloride ([BMIM][Cl]), 1-allyl-3-methylimidazole chloride ([AMIM][Cl]), and 1-ethyl-3-methylimidazole chloride ([EMIM][Cl]), which have proven to be particularly better solvents for cellulose, lignin, and other natural polymers nanotechnical use and stability [80,81].

### 2.9. ILs-Based Membranes

Ionic liquids’ physicochemical properties can be modified to suit a specific chemical application depending on the selection of the appropriate anion, cation, and substituents on the cationic constituent of ILs [82,83]. Although ILs have a very low vapor pressure, they are very sparingly soluble in organic and inorganic compounds [83]. As a result, they can be used directly to make membranes, such as bulk ILs membranes, supported ILs membranes, emulsion ILs membranes, and poly ILs membranes [84]. Based on the modifications, the use of ILs as green/safe solvents in a range of applications, such as separation of various compounds, extractions, absorptions, and chemical reactions have been reported with encouraging results. However, the high cost of preparation, and high energy requirements for recycling, are some of the challenging limitations in using ILs, which have negatively impacted its use, together with its economic viabilities for using ILs in certain potential processes and products. Ionic liquid membrane (ILM) technology has emerged as the new approach to overcome these limitations. Supported ionic liquid membranes (SILMs) are now made up of thin microporous supports with IL-filled pores. Despite their potential use in gas separation applications due to their high permeabilities and selectivity, the SILMs are underutilized due to uncertainty about their mechanical stability under pressure. The ILM techniques have demonstrated superior advantages over their traditional types of supported liquid membranes (SLMs) that are based on organic solvents, thereby requiring a relatively small quantities of ILs or its replacements as a carrier, and do not necessitate any additional steps for ILs recycling. Over recent decades, the use of ILM in the separation process for different compounds have been reported, and a large number of research articles have been published related to this topic [82]. There are three methods in which SILM can be prepared, namely direct immersion, vacuum, and pressure, and all of which can have a significant impact on SILM operational performance due to the relatively high viscosity of involved ILs.

### 2.10. Carbon Nanotubes Hybridizations

Carbon nanotubes (CNTs) are all-carbon inorganic macromolecules that are made up of one or more graphene sheets that are adeptly wrapped into cylindrical shaped tubes, referred to as single-walled carbon nanotubes (SW-CNTs), or multiwalled carbon nanotubes (MW-CNTs) depending on the graphene sheets [85]. The CNTs are promising building blocks for high-performance composites, which necessitate homogeneous dispersion and exfoliation of CNTs to produce individual tubes in the matrix phase. The most common method is to separate CNTs from their bundles using liquid-phase exfoliation and stabilization, before and after functionalization. Thus, the choice of solvent strongly influences, both the dispersion quality, and the functionalization efficiency of the nanotubes. Over the past several decades, ILs have been widely used in synthesis of CNTs hybrid materials, as potential green and designable solvent, linker, and catalyst [85,86]. A synthetic method for carbon hybrid materials consisting of CNTs and reduced graphene oxide (rGO), prepared in presence of ionic liquid-based polymer [poly(ionic liquids), PILs] as a stabilizer and linker, has been reported by Tung and colleagues [87]. The PILs molecules improved the dispersion of CNTs and rGO sheets in the solvents by preventing uncontrolled aggregation, and ILs role as linker facilitated to assemble the two carbons into the nanostructured hybrid material. The complex of CNT/PIL/rGO was formed by the coupling of CNTs and rGO through the PIL-mediated hybridization, in which each component was assembled on the molecular level through electrostatic and stacking interactions. The CNT/PIL/rGO hybrid composites were evaluated for their dual types of sensing applications, one being the chemical sensor, and the other as the temperature sensor. In another study, a simplified method of synthesizing two-dimensional (2D) NiO/CNTs nano-tubular electrode materials using an imidazolium ILs as the reaction medium was reported [88]. In addition, the CNTs are a good carbon material for combination with NiO due to their chemical stability, better conductivity, and large surface area. ILs serve two functions during the synthesis of (2D) NiO/CNTs. First, through conjugate actions between the electrons on the surface of the CNTs and the IL, the dispersion of CNTs is improved. Second, the intramolecular hydrogen bonding between the anion and cation of the IL ensured that the NiO is uniformly distributed on the CNTs’ surface. The electrochemical tests showed that the NiO/CNTs composites prepared in this way have improved the specific capacitance and rate performance of the product.

## 3. ILs and Nano Biotechnology–Current Status, Emerging Trends, and Prospects

ILs property of not crystallizing at room temperature, their viscosity, and other tunable characteristics have opened unsurmountable opportunities in biotechnical and biomedical applications. Applications in biocatalytic enzymatic reactions, immobilized enzymes reactivity, bioreactor applications, the making of biomolecules absorption chip, nanogel and nanoparticles preparations, tissue bioengineering scaffolding, hybridized QDs and nano-biocomposites preparations, and antimicrobial and anti-cancer activity prospects have emerged as part of many different uses of ILs. With the advent of 3rd generation ILs, the specificity of use, and selectivity in applications of ILs based on their designated structural specification led ILs synthesis have formed the new direction for ILs in biotechnical and biomedical fields based on their chemistries development and biological evaluations including the toxicity. Major current applications in these domains are briefly outlined. Future prospects lie in the development and use of polymeric ILs, IL-proteins and other biomolecular conjugates, sub-cellular scale biocatalysis, and the integration of biochemical reactions and purifications/separations as part of the integrated step involving larger molecules.

### 3.1. Nano Bio-Catalysts

Since the early 2000s, the use of ILs in biocatalysis has grown rapidly as part of an effective method in biocatalyzing processes through enzymes stabilization [89,90]. More ILs have been used in enzymatic technology to perform a wide range of catalytic actions. In comparison with the conventional methods, ILs have improved enzymatic catalytic activity, enantioselectivity, and stability. Therefore, ILs have now been recognized as the alternative reaction media for biotransformations [91]. Although most enzymes in ILs have many advantages, a few enzymes still showed lower activity in ILs than in organic solvents. Two approaches utilizing ILs for enzymatic reactions have been explored from the standpoint of biocatalyst facilitating the organic synthesis; namely biocatalysis in ILs, and the enzymes activated by ILs for organic synthesis. Several examples of ILs supported enzymatic reactions for organic synthesis have been known where ILs have been used as solvent, co-solvent. The enzymes, i.e., hydrolases, and oxidoreductases primarily have been utilized in this manner. Su et al. reported a catalytic reaction to produce biodiesel using *Candida rugosa* lipase (CRL) enzyme in IL [92]. After ILs screening, the [Hmim][PF_6_] was found to boost the catalytic efficiency of CRL by 3-folds. Using computational and experimental data, Kim et al. demonstrated the relationship between the enhanced enzyme activity and structural dynamics in ILs [93]. In this study, *Candida antarctica* lipase B (CALB) was used as a model enzyme for efficient biocatalyst for hydrolysis, esterification, and polymerization reactions. Using [Bmim][TfO], *tert*-butanol, [Bmim][Cl], the collected data demonstrated the correlation between the changes in the conformation of the active site cavity, and the enzyme activity which decreased in the following order, [Bmim][TfO] > *tert*-butanol > [Bmim][Cl], thereby demonstrating the superior ability of ILs in enforcing the enzymatic reactions to achieve the biocatalysis.

### 3.2. Bio-Capacitors and Nano-Bio Reactors

The electrochemical stability of electrolytes is considered critical to the working potential of the supercapacitor. ILs are gaining popularity as safe alternatives to current organic electrolytes due to their non-flammability, expanded potential windows, and superior ionic conductivity. The supercapacitors with ILs as electrolytes have high energy density and can be used in a variety of energy storage systems. Previous studies concentrated on electrochemical performance, with only a few exceptions focusing on energy storage process details, or its mechanisms. In supercapacitors, various ILs-based electrolytes, including pure ILs, eutectic ILs, and ILs/organic solvent mixtures, have been extensively investigated. Despite the issues related to the cost and purity of ILs, their benefits as electrolytes are undisputed. Using modified ILs as electrolytes in combination with nanostructured carbon is undoubtedly a promising strategy for developing the next generation of high-energy density supercapacitors with a wide temperature range. A new type of electrolyte in electric double-layer capacitors (EDLCs), which can deliver high energy density due to their excellent physicochemical properties and broad electrochemical window is also being proposed, that can be achieved through the use of appropriately designed and worked out ILs.

### 3.3. Ionic Liquids as Adsorption Chips for Specific Proteins

Since ILs can be modified for specific applications through variation of their chemical structures, their use together with the biomolecules (proteins/enzymes) have been studied over the years [94]. However, the use of pure ILs in certain processes has been associated with certain limitations. The immobilization of ILs onto supports has been reported as one of the alternatives to overcome these drawbacks [95]. In this regard, the supported ionic liquids (SILs) have been successfully used in separation of proteins and enzymes due to their relatively high adsorption and specific recognition capacity [95]. The majority of reported SILs studies in this field used silica, or polymers as the solid phase. The application SILs in proteins separation process is still limited to standard proteins, such as lyz (lysozyme), bovine serum albumin (BSA), bovine hemoglobin (BHb), CYT-c, ovotransferrin (OVT), and hemoglobin (Hb), with limited studies as compared to SILs in the biocatalysis [96]. Using 2-acrylamido-2-methylpropane sulfonic acid imidazole derivative ([AMPS][RIm]) (R: –H, –C_6_H_5_, or –(CH_2_)_3_CH_3_) as the functional monomer, a series of anionic PILs material with high adsorption capacities toward proteins were developed [97]. In this study, the only monomer used to prepare materials for protein adsorption was an anionic polymeric ionic liquid monomer (ILM) containing imidazole cations modified with alkyl derivatives (butyl or phenyl groups) and sulfonic group-functionalized anions. After the characterization of the resultant anionic PIL materials, its adsorption capacity towards proteins was evaluated. The results showed that BSA (842.2 mg g^−1^), and ovotransferrin (OVT, 861.5 mg g^−1^) had a high adsorption capacity at pH 4.0, whereas cyt-c (882.9 mg g^−1^), and BHb (983.4 mg g^−1^) had a higher adsorption capacity at pH 7.0, and casein (605.2 mg g^−1^) had a higher adsorption capacity at pH 8.0. Furthermore, the material was capable of separating BHb from a real bovine blood matrix, demonstrating the relevance of SILs in dealing with complex matrices. In another study, A silane-coupling ILs material, 1-(3-trimethoxysilylpropyl)-3-methylimidazolium chloride ([Tmim]Cl), was used to prepare ILs-functionalized nanoparticles (SiO_2_@ILs) in a one-step grafting reaction [98]. The resulting SiO_2_@ILs was able to adsorb maximum BSA amount of 23.1 mg g^−1^ at pH 7, with electrostatic interactions being considered the main driving force through experimental tests and theoretical density functional theory (DFT) studies.

### 3.4. Interactive Biophases, Nanoparticles, and Nanosystems

Interactive nanosystems may be referred to as smart nanosystems which can mimic various biological behaviors, and physiological functions in a cooperative participation with the host environment [99]. Smart nanosystems are designed as stimuli-responsive systems that undergo chemical and/or physical transitions triggered by temperature, pH, ions, redox, enzymatic, or homeostatic changes in the cells and tissues of the host. The physicochemical properties of ILs, and their flexibility for modifications, position them as interesting candidates for designing the building blocks of stimuli-responsive nanosystems by chemically modifying and/or blending ILs with nanomaterials to obtain thermo-responsive, pH-responsive, ion-responsive, and electro-responsive behaviors [100,101]. Before ILs can be harnessed for smart properties, a thorough understanding of ILs–biomaterial interactions, and the chemical and physical alterations in IL structures within the nanosystems are required. This will allow the development of multifunctional smart nanoparticles and nanosystems for sensing, diagnostic, and drug and gene delivery applications, wherein two or more nanosystems operate via stigmergy (where the action of one nanosystem stimulates the action of another nanosystem) to induce a biological or physiological change in the local environment of the targeted, or the diseased tissues [102]. Recent ILs-based nanosystems research had focused on materials characterization, such as the study of thermo-responsive ILs (imidazolium cation-based) nanostructured hydrogels for the development of stimuli-responsive macromolecular nanosystems to control solubilization, dispersion, transport, and shuttling of molecules and nanoparticles [103]. Pharmaceutical applications include selective drug delivery using pH-responsive boron phenylalanine targeted nanoparticles based on ILs-modified chitosan [104], whereas the development of fluorinated ILs-based nanoprobes were studied for their sensing and multicolor imaging of multiple biological targets [105]. Abiotic–biotic communication, based on orchestrating chemical communication (via molecules) between nanoparticles and living cells, is an emerging, but barely studied, topic in nanobiotechnology [102]. For a new level of functionality and complexity, nanoparticles and nanosystems need to be designed to interact with the living environment of the host organism through interrogating, sensing, signaling, and autonomously reacting to diagnose a disease, or deliver therapeutic molecules [99]. This level of bioinspired interactive function has called for a shift from developing ‘smart’ stimuli-responsive nanosystems to engineering ‘intelligent’ nanosystems that can analyze, regulate, and perform complex functions amongst living host cells, and the report of its early development [106]. Abiotic–biotic chemical communications with the development of intelligent nanosystems have the potential to open new insights into the design, functional variability, and use of personalized nanomedicine.

### 3.5. Tissue Bioengineering Scaffolds, and Bone-Graft Materials Development

Due to various bone diseases, such as bone infections, bone tumors, and bone loss because of trauma, there is a growing need for bone regeneration [107]. Autografts, allografts, xenografts, and other substitutes, such as metals, synthetic cements, and bio-ceramics are currently being explored to be used to treat bone defects. However, these alternatives are far from ideal, and each has its own sets of issues and limitations. As a result, current research has focused on bone tissue engineering, in which a 3D porous scaffold is loaded with specific living cells and/or tissue-inducing factors to initiate tissue regeneration and/or replacements in natural settings [108]. A large number of natural and synthetic materials of various types have been evaluated for their potential as scaffolds for tissue bioengineering to create support structures for three-dimensional tissue formations [109]. Furthermore, the bone tissue bioengineering products intended for scaffolding materials should be osteoconductive so that osteoprogenitor cells can adhere and migrate on the scaffolds, further differentiate, and, finally, form the new bones. Despite the outstanding therapeutic benefits, these materials lack solubility in water and other common biocompatible organic solvents [110]. In this regard, ILs are thought to be potential substitutes for environmentally friendly green solvents in a long-term process [111]. Cellulose is one of the polymeric materials that have found applications in tissue bioengineering that is capable of mimicking the natural bone features to certain extents. Swatloski et al. reported in 2002, that cellulose could be dissolved in ILs, 1-*n*-butyl-3-methylimidazole chloride, and 1-*n*-butyl-3-methylimidazole chloride (BmimCl) [112]. Zhang et al. discovered in 2004 that 1-*n*-allyl-3-methylimidazolium chloride (AmimCl) was an effective cellulose solvent [113]. Recently, cellulose–hydroxyapatite nanocomposite scaffolds were successfully prepared using AmimCl solution, showing a better dispersion of hydroxyapatite in cellulose as compared to other solvents. The osteoblast cells were used to perform in vitro biological evaluations on the samples and AmimCl was recycled [114]. The growing number of literature reports on tissue bioengineering for developing appropriate scaffolds using ILs have indicated that ILs application in the field is gradually increasing.

### 3.6. Applications in Genes, and Other Cellular and Sub-Cellular Deliveries

The processing of nucleic acids with ILs is an emerging idea in recent times. Though the technique is relatively new, the application of ILs in gene delivery has been prominently established. However, an increasing number of studies on ILs in intracellular nucleic acid delivery have emerged in the last few years. In particular, ILs have been successfully tested as stabilizing agents for DNA storage [28]. ILs can also easily undergo intermolecular interactions with a range of polar/non-polar solutes, including biomolecules, such as proteins and DNA [29]. A recent study used nucleobases in ILs, [C_2_Mim][OAc], where the authors solvated uracil in ILs to promote its dissolution [115]. In another study, authors developed a lipid-like ILs in which the preparation contained 2-mercaptoimidazolium, and 2-mercaptothiazolinium head groups tethered to two long saturated alkyl chains. The developed carriers were used as an in vitro delivery system for plasmid HEK DNA into 293T cells [43].

### 3.7. Micellar Systems, and Nanogels for Biotechnical Applications

Micelles are self-assembling nano-sized colloidal particles with a hydrophobic core and hydrophilic tails, and are currently being successfully used as carriers for water-insoluble drugs in the field of pharmaceutical nanotechnology. Polymeric micellar systems possess high stability, both in vitro and in vivo conditions. As already exhibited, the ILs have better biocompatibility, and solubilize several poorly water-soluble drugs. The drug-loaded micellar systems, prepared through intermediacy of ILs, are under constant investigation in different stages of preclinical and clinical trials [116]. Nanogels are nanoscale hydrogels composed of a three-dimensional network of polymers, with a greater ability to absorb large amounts of water through their system. Nanogels have unique physiochemical properties and have merits over most of the NPs in drug delivery applications. Polysaccharides have been reported as the widely used self-assembled nanogel biomaterials fit for use in drug delivery due to their excellent biocompatibility, and ease of functionalization [117].

### 3.8. Cancer Hyperthermia and Ionic Liquids Role

In oncology, hyperthermia therapy (or thermal therapy) is the controlled heating of tumor site to temperatures between 40 °C and 43 °C. Heating tumorous tissues above the normal physiological temperature of 37 °C initiates the breakdown of proteins and enzymes, together with it increases blood perfusion. Since cancer tumors have low blood perfusion and low pH, these parameters have been used as effective triggers for multifunctional stimuli-responsive nanomaterials for targeted drug delivery and diagnostics. Thus, locally heating the tumor site increases blood perfusion which provides a stimulus for enhanced thermo-responsive drug delivery of nanocarrier loaded chemotherapeutic agents. Combined with thermal therapy, thermo-responsive nanocarriers (such as polymeric nanoparticles, polymer micelles, core–shell nanoparticles, and liposomes) increase drug accumulation within the tumor and decrease systemic toxicity [118]. In addition to its anticancer potential, ILs paired with polymeric nanoparticles can offer improved functionality in cancer drug delivery applications. In one study, complexing poly(IL-co-N-isopropylacrylamide) with deoxycholic acid produced dual pH- and thermo-responsive nanoparticles for enhanced doxorubicin delivery at an acidic pH of 5.2 and elevated temperature of 43 °C [119]. In another study, hollow polydopamine nanoparticles loaded with an ILs (of high microwave irradiation susceptibility) and doxorubicin was developed for combined microwave thermal therapy and chemotherapy in cancer hyperthermia [120]. Recent research in cancer-hyperthermia drug delivery focuses on merging the features of magnetic nanoparticles with pH-responsive, thermal-responsive and/or redox-responsive polymers for designing synergistic multifunctional smart nanosystems for drug delivery purposes [121]. Magnetic iron oxide nanoparticles are incorporated into stimuli-responsive biomaterials (such as thermo-responsive hydrogel nanocomposites) or functionalized with stimuli-responsive polymers (such as bioreducible and pH-degradable polymer coatings) that respond to changes in heat, pH, and redox homeostasis [122,123,124] have been developed. These magnetic nanosystems are exposed to alternating amplitudes of magnetic field strength and frequency to generate heat and induce hyperthermia in the tumor region—a process called magnetic hyperthermia [125]. A recent study revealed that iron oxide nanoparticles modified with polyethylene glycol and ILs, e.g., *n*-hexylpyridinium bromide resulted in a fast and high loading capacity of cisplatin [126]. Therefore, combining IL-decorated magnetic iron oxide nanoparticles, SPIONs (Super paramagnetic iron oxide nanoparticles), the magnetic hyperthermia and magnetic fields-directed therapy with stimuli-responsive controlled drug release have the potential to result in powerful multimodal treatment strategies for cancer treatment.

### 3.9. Fluorescent Hybridized Quantum Dots- Preparation and Uses

Due to their low cost, tunable multicolor luminescent properties, biocompatibility, low cytotoxicity, and hydrophilicity, the CNDs (carbon nanodots, also known as carbon QDs) are considered superior to semiconductor quantum dots, which exhibited high cytotoxicity and poor hydrophilicity [127], have been well adopted for the purpose. In addition to mediating the physicochemical properties of carbon nanodots, ILs are key adjustors of its fluorescent properties [106]. Imidazolium-based ILs are an important class of ILs due to their biodegradability, non-toxicity, and ability of the imidazolium ring to bond to metals as ligands and form hydrogen bonds with proteins and drugs [128]. Thus, through the years, imidazolium-based ILs have been the focus of IL-functionalized quantum dots for ion sensing and fluorescent inks in biosensing and bioimaging applications, respectively [129]. Utilizing the fluorescent properties of QDs and the protein affinity of ILs, one study synthesized silica-capped imidazolium-based IL-CdTe QDs possessing selective recognition and fluorescent detection of hemoproteins through ILs-protein covalent interactions in biological fluids [128]. The imidazolium-based ILs-functionalized CNDs have been widely studied for their sensitivity and selectivity for metal ions (Fe^3+^, Cu^2+^, Hg^2+^) in fluorescent biosensing for environmental and biomedical applications, respectively [106,127,130]. The ion sensing of metal species is enabled by the fluorescence quenching of ILs-CNDs complex by various metal ions [106]. The nitrogen-containing bulky cation found in most ILs makes them ideal precursors for luminescent carbon nanodots [130]. This makes the bioimaging applications of ILs-functionalized CNDs an important development for non-invasive analytical techniques [106]. Compared to IL mediated fluorescence, traditional organic dyes are also easily photo-bleached and, thus, are unsuitable for long periods of bioimaging applications [106]. Due to its biocompatibility and versatility, the CNDs functionalized with ILs can be made hydrophilic, hydrophobic, or organophilic in nature. A study indicated that the longer side chains of cations in imidazolium ILs, and the weakly nucleophilic anions tend to produce high fluorescence in ILs-functionalized organophilic CNDs [131]. A recent study showed that hydrophobic ILs-carbon nanodots (CNDs), prepared through solvothermal methods and interaction with BSA, exhibited improved fluorescence compared to its hydrophilic counterparts [132]. Thus, the synthetic parameters of carbon nanodots and the chemical structure of ILs can be modified to influence the fluorescent properties and bioimaging capabilities of these unique analytical probes.

### 3.10. Nano Ion Channels for Biological Uses

**Figure 7:**
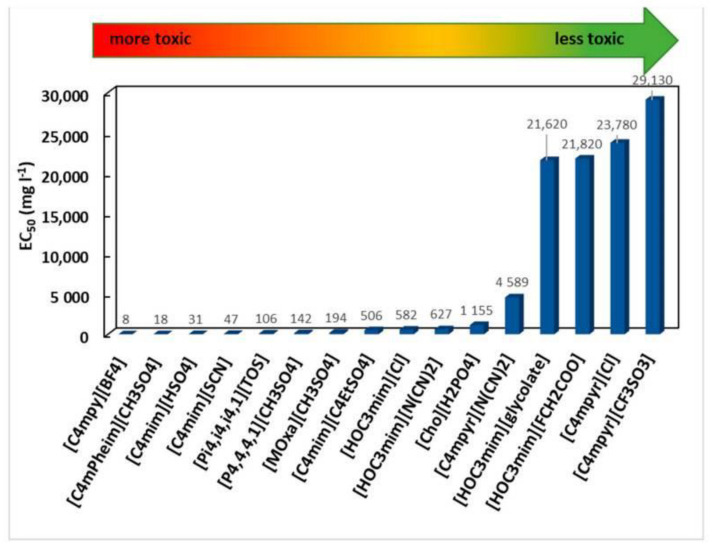
EC_50_ values for ILs towards A. fischeri studied by Hernández-Fernández et al. Reprinted with permission from Ref. [136] 2021, Creative Commons CC BY4.0. Original data obtained with permission from Ref. [137]. 2015, Elsevier.

It was believed for a long time that ILs were safe for the environment because they are composed of charged units. However, ILs exhibit high thermal stability and low vapor pressure at normal temperature and were, thus, considered non-volatile, as in, they do not evaporate in the atmosphere [133]. In recent years, ILs have been considered a promising alternative to toxic organic solvents. The possibility of the release of ILs into the environment is growing systematically. There is an increasing need to determine the toxic and antimicrobial influences of ILs on the environment. In recent years, many bioassays were conducted to evaluate the eco-toxicity and biodegradability of ILs [133,134]. The “green” features of ILs were the most discussable question since it has a toxic effect on various organisms [133]. There are several factors that contribute to the toxicity of ILs, but cholinium-based ILs are generally the least toxic. However, as their side chain length, hydrophobicity, anion lipophilicity, and instability increases, so does their toxicity [135]. The toxicity data of 16 ILs were reported by Hernandez-Fernandez et al., using the *A. fischeri* bioassay, in an effort to establish guidelines for designing more environmentally friendly ILs (Figure 7) [136,137]. Therefore, developing new biodegradable IL-composites with less toxicity is a need in the current times [133]. The basic principles of green ILs are that they should be safe for the environment and should not liberate any hazardous substances capable of killing living organisms. Several studies have been reported that concern the assessment of ILs toxicity on the lactic acid-producing bacteria, *Vibro fischeri, Escherichia coli, Pichia pastoris, Bacillus cereus*, and others [134,138,139].

### 3.11. Nano Bio-Composites and ILs

Bio-based pharmaceutical products have gained increased interest since the issue of sustainable development and environmental concern were raised. Consequently, biopolymers have been widely used in the pharmaceutical field to formulate nano-sized fillers to improve physicochemical properties, such as stiffness, permeability, crystallinity, and thermal stability, and, thus, resulting in the emergence of ‘nano-bio composites’ [140]. A recent review article reported the developments of new promising composites of ILs with inorganic oxide nanomaterials, wherein the study synthesized nanocomposites of physical ILs mixtures with inorganic oxides and porous nanocomposite oxide materials were reported. In the study, ILs could be included in the nano-cavities of the inorganic materials [141]. In another study, ZnO-nanocomposites coated with benzimidazole-based ILs were prepared (ZnO@IL). The authors mentioned that ZnO@IL showed antibacterial behavior and the developed nanocomposite displayed significant microbial inhibition of approximately 45%. In addition, the authors observed a considerable decrease in bacterial cell growth with ZnO@IL treated culture medium [142]. Another recently reported study prepared a polymeric nanocomposite system consisting of PILs, polymethylmethacrylate (PMMA), and MWCNTs as fillers, where ILs functioned as a plasticizer and dopant for the MWCNTs. The prepared nanocomposites exhibited variable mechanical and conductive properties [143]. These properties can allow for the development of 3D-printed robotics, electromechanical sensors, electrochemical systems, and flexible devices that can be easily fabricated in a mold-free and entirely automatic process.

### 3.12. Drug Development: Bioavailability, Bioactivity, and Cellular Interactions

Several studies have shown that the therapeutic efficacy of 40% of commercially available drugs and 90% of those under study is severely compromised by inadequacies in their pharmaceutical properties [144]. This includes poor solubility and bioavailability, stability problems, and many other drawbacks [144,145]. In order to enhance the delivery of such drugs, there is a strong demand for new strategies to achieve an improved drug biopharmaceutical performance, take a drug to the molecular level in vivo, and do so in a biocompatible and non-toxic manner. In addition to their interesting properties alone, ILs are also of particular interest in this connection when combined with drug delivery systems [145].

Many studies have tried to develop ionic liquid-based systems to deliver and enhance the efficacy of the therapeutic agents, APIs, and other drug materials. The transdermal delivery of several small and large molecules have been enhanced using the choline-based ILs, particularly choline and geranic acid (CAGE). Although ILs for transdermal drug delivery have been extensively studied, detailed studies are still required. In a previous report, authors studied naked DNA intradermal injections for efficient and low variability gene transfer into skin cells. The study achieved a higher level of gene expression for naked DNA with isotonic phosphate-buffered solution [146]. In another study Eden et al., studied the role of anion and cation properties in IL-mediated transdermal drug delivery [147]. Using two model drugs with different hydrophilic properties, acarbose, and ruxolitinib, as well as 16 ILs, the dependence of the skin penetration was evaluated. Through the obtained experimental data, it was demonstrated that the effectiveness of ILs in enhancing transdermal drug delivery was inversely related to their inter-ionic interactions. Therefore, the author hypothesized that modification of both anion and cation with reduced interionic interactions and their combination could yield novel ILs for enhanced delivery of drugs of different hydrophilicity. ILs role in understanding of gene functions, gene therapy through ILs assisted gene delivery, and development of molecular medicine were proposed through model studies on eukaryotic cells. The micro-structuring and tunable properties, lends ILs suitable for gene delivery and studying the interactions of ILs with the nucleic acid components [28].

## 4. Environmental and Animal Toxicities: Aquatic, Terrestrial, and Biosphere Contaminations, and Risk Assessments

The notion of ILs being green and environmentally friendly is not supported by toxicological studies. ILs toxicity is manifested in terrestrial and aquatic conditions. The water solubility, and negligible vapor pressure, has, together, played a role, since their non-volatility has prevented their direct release into the atmosphere. However, their growing use in industries, including pharma industries, has opened them as a threat for contamination. ILs interaction with the components of biosphere needs prioritized toxicity studies and related risk assessments. ILs microbial degradation, the materials sorption and desorption, in aquatic media and earth spaces are plausible points of ILs contaminations. Risk assessments related to the consequences of ILs use and subsequent contamination have been considered [148,149]. Bioassays to evaluate biodegradability, material retention, eco- and cyto-toxicities, and antimicrobial influence on the surroundings are important and have been worked out in experimental and in silico conditions, utilizing the structures and properties contributions to projected toxicity through QSAR/QSPR (Quantitative Structure Activity Relationships/Quantitative Structure Property Relationships) for designing and developing safer ILs. A new genre of biodegradable ILs derivatized from amino acids, sugars, choline, and bicyclic monoterpene moiety have also been produced [133,150,151]

Among the main drivers of toxicity, the lipophilicity is of prime concern, thereby the role of cation and its structure and the influence of the chain and its length are important. The anionic part plays minor but not negligible role in elicitation of toxicity [136,152]. The poor biodegradability is another factor contributing to the toxicity [153,154]. The biosystems and its toxic components owing to ILs have been recorded. Many enzymes exhibit enhanced bio-catalytic activity, stability, and selectivity. ILs are also capable of dissolving a broad range of substrates for the purpose. The potential risks of ILs on the enzymatic activity and stability have been worked out [155]. A comprehensive database on ILs toxicity with over 4000 pieces is available [151]. Based on the dataset, QSAR analysis was performed to predict the structural pattern responsible for the toxicity elicitation. ILs toxicity towards the Leukemic rats’ cell lines, IPC-81, was developed to further study the structure factor’s contribution to toxicity (EC50 values). The heuristic method (HM) for multiple linear regression (MLR) and support vector machine (SVM) were used. The SVM model was found comparatively better to predict toxicity, and the study found that increasing the O atom abundance led to decrease in the toxicity.

The acute and sub-chronic oral toxicity of dodecyl dimethyl ammonium saccharinate [DDA][Sac] was evaluated, which varied from 1.44 mM to 5.47 mM. Exfoliation of colon surface layer and alveolar septa in lungs parenchyma in acute toxicity tests, and for 28 days, subchronic toxicity tests at 10, 30, and 100 mg/kg/day of [DDA][Sac] were observed. High dosed rats showed reduced food consumptions and reduced body weight gains were observed. Mild hematological changes were observed in mid-dose female rats. The LD50 cut-off was at 500 mg/kg. An increase in the ALT, SDH, and ALP levels, and increase in GGT activities, together with increase in glucose, blood urea nitrogen, and creatinine concentrations were observed. The changes were not common for both the genders. The lowest adverse effects level was determined to be observed at 10 mg/kg/day.

The DNA damage and other biochemical toxicity of imidazolium-based ILs with several anions in soil on *Vicia faba* seedlings have been assessed [156]. Imidazole nitrates-based ILs up to C8 carbon chain length at cut-off 1-octyl-3-methyl imidazole showed gradual increase in acute toxicity (LC50) in earthworms, *Eisenia foetida*, although the toxicity was observed till C12 carbon chain length alkyl chain of the cationic part of the structure [157].

## 5. Conclusions, and Future Directions

The synthesis of ILs on a large scale is improving day by day due to advancements in the scaling up of preparation processes’, and modifications in the synthetic protocols of ILs, as well as due to their structural variation possibilities in the anionic and cationic entities. As part of the improved productivity, the cost effectiveness of ILs is pertinent to be achieved. ILs use in different nanotechnical, especially in the nanobiotechnology fields is expected to rise exponentially. The nanoparticles-ILs combinations are recent important and appealing nanomaterials developed for various applications in pharmaceutical drug development and biotechnology, and are expected to get further impetus through introduction of ILs use at larger scales in the industrial sector. However, the toxicity of ILs on human exposure and the environment still needs to be explored further to implement its safe and efficient use for future, although initial lines on toxicity evaluation, prediction, and database preparation have been achieved. Areas needing further work are the purity controls, toxicity removal at eco- and cyto-levels, and development of completely biodegradable, biocompatible ILs that are important for clinical use. ILs as true designer solvents with a definite selection of structural components for synthesis, to yield ILs that are fit for use in the desired application, are of primary focus. Developments in chemical synthesis, purity control processes, and technique development, and manufacturing of bulk-scale and highly pure ILs are significantly required to produce IL materials fit for biological applications that meet the regulatory requirements.

## Figures and Tables

**Table 1:** Applications and functions of commonly used ILs.

Application Area	Function(s)	Commonly Used ILs	References
Nanotechnology Nanoparticles and nanocrystals Quantum dots Nano-structured coatings and films Nanoporous materials	Control of aggregation, crystal growth, and particle size and shape Thermal stability and antioxidant properties Plasticizing effects Porogenic solvent	Imidazolium-based ILs (considered biodegradable and non-toxic) Alkyl ammonium ILs	[9,10,11,12]
Green chemicals Solvents Additives Nano biocatalysts	Replacement of volatile solvents for dissolving organic and inorganic compounds Stabilizers, emulsifiers, and surfactants Improve enzyme catalytic activity and stability	1-butyl-3-methylimidazolium thiocyanate 1-Butyl-3-methylimidazolium hexafluorophosphate 1-Hexyl-3-methylimidazolium hexafluorophosphate 1-octyl-3-methylimidazolium hexafluorophosphate Imidazolium-based ILs	[13,14,15]
Drug delivery and biomedicine Drug delivery vehicles Therapeutic agents Micellar systems and nanogels Tissue engineering scaffolds and materials	Control solubilization, dispersion, penetration, and transport of molecules Antimicrobial properties Anticancer properties Adjusting biomaterial amphiphilicity Biomaterial preparation for polymer dissolution and processability	Imidazolium-based ILs Imidazolium, pyridinium, pyrrolidinium, piperidinium, ammonium ILs (potential environmental toxicity to beneficial microorganisms) Imidazolium, phosphonium, ammonium and guanidinium ILs (non-selective toxicity between normal and carcinoma cell lines) Imidazolium-based ILs Choline-based ILs Imidazolium-based ILs (enhanced biocompatibility and non-toxic for biomedical applications)	[5,13,16,17]
Biotechnology Extraction and separation technologies Nano-capacitors and bioreactors	Extraction of antibiotics from fermentation broths Extraction of metal ions Protein separation Electrolytes	Imidazolium-based ILs (considered biodegradable and non-toxic) 1-Ethyl-3-methylimidazolium bis(trifluoromethylsulfonyl)imide 1-Butyl-3-methylimidazolium bis(trifluoromethylsulfonyl)imide Alkylimidazolium ILs Imidazolium-based ILs Choline-based ILs Cations: imidazolium, tetraalkylammonium, pyrrolidinium, piperidinium and pyridinium Anions: bis(trifluoromethane sulfonyl)imide (TFSI^-^), bis(fluorosulfonyl)imide (FSI^-^), hexafluorophosphate (PF6^-^), tetrafluoroborate (BF4^-^), and Cl^-^	[18,19,20,21,22]

**Table 2:** Physical properties of ILs used in separation technology.

Chemical Formula		Abbreviations	Melting Point, °C	Density (g/cm^3^), 25 °C	Viscosity (cP), 25 °C	Molecular Weight	References
Cation	Anion						
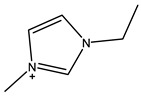	[BF_4_]^−^	[EMIM][BF_4_]	6	1.248	66	197.8	[7,8,70]
	[PF_6_]^−^	[EMIM][PF_6_]	58–62	1.373	450	256.13	
	[BF_4_]^−^	[BMIM][BF_4_]	−82	1.208	233	225.80	
	[PF_6_]^−^	[BMIM][PF_6_]	10	1.373	400	284.18	
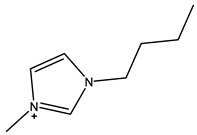	[Br]^−^	[BMIM]Br	60	1.134	Solid	218.9	
	[Cl]^−^	[BMIM]Cl	89	1.120	Solid	146.50	
	[CF_3_SO_3_]^−^	[BMIM][CF_3_SO_3_]	16	1.290	90	260.0	
	[(CF_3_SO_2_)_2_N]^−^	[BMIM][(CF_3_SO_2_)_2_N]	−4	1.420	52	487.9	[23,73]
	[NTfO_2_]^−^	[(CF_3_SO_2_)_2_N] [BMIM] [NTfO_2_]	−8	1.404	48	433.0	
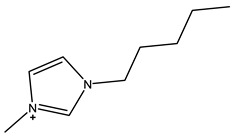	[BF_4_]^−^	[AMIM][BF_4_]	−88	1.231	321	240.02	[74]
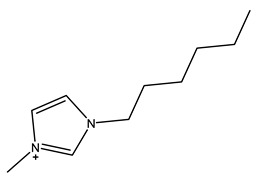	[BF_4_]^−^	[HMIM][BF_4_]	−82	1.075	211	254.08	[75]
	[PF_6_]^−^	[HMIM][PF_6_]	−61	1.304	800	312.00	
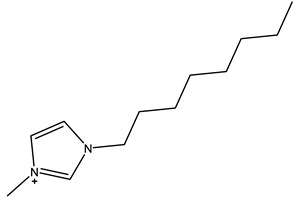	[BF_4_]^−^	[OMIM][BF_4_]	−79	1.11	440	281.8	[76]
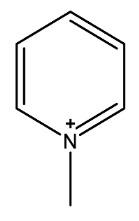	[Cl]^−^	[OMIM][Cl]	0	1.000	16,000	230.50	[77]
	[NTfO_2_]^−^	[MPPyr] [NTfO_2_]^−^	0	1.44	39	416	
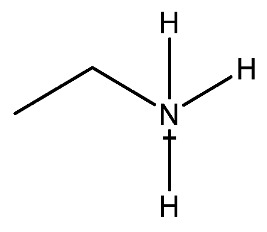	[HCOO]^−^	BAF	−10	0.99	11.5	91	
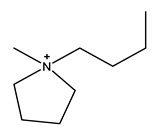	[NTfO_2_]^−^	[BMPyrrol] [NTfO_2_]	−50	1.4	71	422	[21]
